# The experience of puberty in Iranian adolescent girls: a qualitative content analysis

**DOI:** 10.1186/1471-2458-12-698

**Published:** 2012-08-27

**Authors:** Nayereh Azam Hagikhani Golchin, Zeinab Hamzehgardeshi, Moloud Fakhri, Leila Hamzehgardeshi

**Affiliations:** 1 Department of Midwifery, Islamic Azad University, Gorgan branch, Gorgan, Iran; 2 Department of Midwifery, Mazandaran University of Medical Sciences, Sari, Iran; 3 Mazandaran University of Medical Sciences, Sari, Iran; 4 Department of Health Services Administration, Science and Research Branch, Islamic Azad University, Fars, Iran

**Keywords:** Iranian adolescent girls, Qualitative study, Sexual orientation, Experience of puberty, Content analysis

## Abstract

**Background:**

Adolescence is an important stage in human life span. Physiologic changes associated with puberty manifest themselves in often complex and bizarre ways to which girls show different reactions. This study aims to explore to puberty experiences in adolescent girls who live in the city of Sari in Iran.

**Methods:**

The present study is a qualitative study of content analysis. Sampling took place in the city of Sari, Iran and was objective focused in accordance with qualitative studies. Participants were 38 girls of 12–20 years old who had at least experienced 3 menstrual cycles. Data was collected by means of focus group and in-depth interviews.

**Results:**

As follows, Seven main themes were extracted from the interviews are follows: Menarche as the most unpleasant event in puberty, getting nervous about and ashamed of bodily changes, psychological changes, discordance with parents, sexual orientation and the need for education on this issue, scholastic dysfunction and religious considerations.

**Conclusion:**

The results showed that for the majority of the participants puberty was an unpleasant experience. Most of them were in need of education on how to go about the issues surrounding puberty. The society, families and of course the adolescents themselves are responsible to work together in order to create an atmosphere in which correct information on puberty and the associated issues are readily accessible.

## Background

Adolescence is an important stage in the human life span [1] marked by the onset of puberty. It is a stage in which adolescents cross the line between childhood and adulthood [2]. Today, adolescents represent an independent entity in world healthcare systems [3]. This has especially been the case since the International Conference on Population and Development in 1994 [4].

Based on results of the Iranian national census, in a population of almost 70 million in Iran, some 11.5 million were girls 10–24 years old. In Iran, girls’ health status is often considered of greater importance than that of boys. There are several cultural and social reasons for this point of view. First, a girl’s lifespan includes several stages and milestones such as infancy, childhood, puberty, marriage, pregnancy, childbearing and menopause. Furthermore, puberty has challenges that can affect not only the future of a girl but also that of her children. Second, in many societies girls are the first to sacrifice in response to cultural problems [5]. Although they are not yet fully equipped with adaptive skills such as problem solving, they are confronted by a host of personal and social concerns associated with puberty that if not addressed can lead to adverse consequences for the next generation. Thus, healthy mothers form the basis of prosperity and wellbeing of any society [1].

Physiological changes associated with puberty often manifest themselves in complex and unique ways, and girls can have a variety of reactions to such changes [5]. The results of a study on 191 Turkish adolescent girls showed that although 52.9% of them considered menarche a natural phenomenon, for the remainder it represented a terrible experience [6]. Another study in America reported that there is a significant difference between black and white girls in terms of their negative feelings towards puberty [7].

Menarche is associated with a sea of biological changes that encompass physiological, sexual, emotional and cognitive aspects and may lead to pubertal mood disorders. It is not just hormones that are behind mood disorders in teenagers. Other factors such as changes in socializing pattern, conviction, and vision as well as teenagers’ altered understanding of themselves may be the culprits [8]. Puberty can be a challenging time that is often associated with early sexual activity, dropping out of school, substance abuse, unsuccessful marriage, unwanted pregnancy, sexually transmitted diseases (STDs) and psychosomatic and social problems [5,9]. In addition, the lower average age at menarche in industrialized countries throughout the 20th century has resulted in females becoming fertile before fully acquiring the necessary psychological capacity to adjust. Studies have shown the influence of social and cultural factors on pubescent behavioral changes and thus the need for studies in different cultural and socioeconomic environments [9,10].

Because of traditional cultural restrictions on discussing sexual and reproductive health and related issues, many girls in our country lack appropriate and sufficient information regarding puberty and menstrual health. This can result in incorrect and unhealthy behavior during their menstrual period. In Iranian culture, menstruation and health during puberty is rarely discussed at home or school [11], although in recent years, reproductive health care has been a primary concern of the Ministry of Health and Medical Education and the Ministry of Education in Iran. In this regard, the Department of Youth and School Health started a reproductive health care program. However, sexual and reproductive health education has not been clearly and completely provided via schools, mass media or families [11]. In addition, one study suggested that reproductive health education initiatives that involve mothers may be most appropriate in terms of cultural and religious sensitivities, and are in keeping with the wishes of the girls themselves [12].

Based on Islamic rules, there are several prohibitions for a menstruating woman. She cannot enter a mosque. In addition, she cannot pray or fast during Ramadan, is prohibited from daily prayers and cannot touch the Quran. Furthermore, she is not allowed to have sexual intercourse during menstruation and must complete a ritual washing after bleeding. Only then is she able to perform prayers, fast and enter the mosque [13].

In view of the fact that the bulk of literature on adolescent health-related issues, regarding girls in particular, has been written by adults, a paucity of information from adolescents themselves exists. It is possible to deepen our insights into adolescents’ problems and educational needs by exploring their own ideas and experiences and taking measures to address them. We found that such a probing and qualitative approach in Iran, and in Sari in particular, is unprecedented. Therefore, this study was conducted to investigate adolescent girls’ own opinions regarding pubescent experiences in an effort to acquire basic information needed to implement suitable interventions aimed at improving their health status.

## Methods

### Study design and participants

The current study is a qualitative content analysis. Participants who met the inclusion criteria were girls 12–20 years old from the city of Sari who had experienced at least 3 menstrual cycles. They were enrolled using a subjective sampling method (appropriate for qualitative studies) that continued until the point of data saturation (a total of 38 participants). Initially, purposeful and snowball sampling methods were used for data collection.

Data were collected by semi-structured interviews. Once the participants were informed of the objective of the study and consent to participate was obtained, they engaged in a focus group discussion (FGD) and individual in-depth interviews that took place in venues convenient for them (schools, universities or their homes). Initially, six focus groups were conducted. Five girls participated in each FGD. In addition to the FGDs, individual in-depth interviews were conducted with eight girls who were too embarrassed to participate in a focus group. They preferred to explore their experiences in a private session. All FGDs and individual interviews were conducted confidentially. Finally, researchers extracted a number of themes and categories by analyzing qualitative data.

### Data credibility

The credibility of the research was checked. The following were performed to ensure rigor and trustworthiness in this qualitative inquiry. First, a member check procedure was used, and primary codes were considered for the participants’ responses. The research team members (internal check) evaluated the interviews, codes and derived categories to assess the accuracy of the coding process and to determine whether they applied similar codes. The transcripts were reviewed by the participants. In this step, the interviewer’s relationship with the participants further developed, with more interview sessions conducted to assure data accuracy. Methods such as prolonging engagement, devoting enough time and developing a good relationship were used. Additionally, a peer debriefing method (external check) was used in which the transcripts and findings from the data were given to experts for their comments, non-correlated occasions were omitted, and comments were added to confirm the results obtained. In this step, the experts' comparison of the study analysis with correlated studies and receipt of their feedback increased the quality and precision of the analysis [14].

The ethics committee of Mazandaran University of Medical Sciences (code number: 89-10-29-89150) approved the protocol of the study. All participants were informed of the purpose and methods of the research. In line with ethical considerations pertaining to qualitative studies, the researchers assured confidentiality and anonymity, preserved the participants’ right to withdraw at any time during the study and obtained informed consent from participants and their parents. Participants were initially asked for demographic information including age, age at menarche, amount of income, level of education and parents’ occupation. Participants were then asked to provide their opinions about the experience of puberty. To assist with the interview process, general interpretive questions with open answers were designed to create a semi-structured interview, with the answer to each question leading to the next question. Questions discussed in focus groups and in-depth interviews included the following:

· What are your feelings about the experience of puberty?

· What puberty issues did you experience?

· What caused these problems? Please explain more about these problems.

· What are your pubertal concerns in order of priority?

All interviews were recorded, transcribed verbatim and analyzed. The duration of FGDs was between 1 and 3 h, with individual interviews averaging 60–90 min. The interviews continued until the point of data saturation.

### Analysis

Data analysis was conducted to extract codes, categories and themes by qualitative content analysis. Qualitative Content analysis is defined as the process of organizing qualitative data regard to emerging categories, concepts, and themes.

In current study qualitative content analysis was conducted as follow:

1- Initially, all interviews were transcribed verbatim.

2- The narratives were identified by review interview transcripts. Then categories were formed after doing coding.

3- Interview transcriptions, all cods, and categories were reviewed several times. Finally, the meaningful themes were emerged by comparison of the different individual’s opinions and experiences about puberty [9,14].

### Results

The number of participants by age group was as follows: 13 years old (13), 17 years old (11), 18 years old (4), 19 years old (4) and 20 years old (6), for a total of 38 girls. Thirteen participants were guidance (lower secondary) school students, 1 was a high school student, 4 were college students and 10 were university students. Age at menarche ranged from 10 to 15 years, with a mean of 12.6 years.

Seven main themes were extracted from the interviews: menarche as the most unpleasant event in puberty, nervousness and shame regarding bodily changes, psychological changes, and discord with parents, sexual orientation and the need for education on this issue, scholastic dysfunction and religious considerations.

#### Theme 1: Menarche as the most unpleasant event in puberty

The majority of participants named menarche as the most unpleasant pubertal event. This theme included numerous codes such as hatred, fear, shame, surprise and a kind of sickness. One of the participants described her experience this way:

“When I menstruated the first time, I was shocked. I didn’t think it would be like that. I had a terrible feeling. It was absolute misery. Although I let my mom know about my first menstruation, I avoided her because I was too embarrassed to talk about the issue with her.” (13-year-old girl)

The experience was perceived differently in girls with early and late onset menarche. Two participants with late onset menarche (after 16 years old) stated that menstruation relieved their worries.

“I was concerned about the delay in my menstruation. I feared I had a problem, and I would be barren in the future. My mom was also worried, and my menstruation relieved both of us.” (20-year-old girl)

One of the participants who was 10 years old at the time of her menarche stated the following:

“I absolutely had no idea about menstruation. I was shocked to my foundation. I didn’t know it was a sign of growing up; I assumed it was a sickness. I didn’t dare divulge it to my mom until she realized for herself. I was also afraid I would be ridiculed at school if my friends found out.”

The majority of the participants made reference to unreliable and vague sources of information, including mothers and friends. For example, some adolescents reported that sources of information about menstruation and answers to their questions about personal health were not adequate. A participant who was 16 years old shared this:

“I'm wondering because I got my menstrual knowledge from my mother and friends, just in time, I think. Almost all was inadequate, and I did not see any scientific validity.”

#### Theme 2: Nervousness and shame regarding bodily changes

Sensitivity to appearance was reported by the majority of participants.

*“I stand in front of the mirror for hours and check my clothes and appearance.” (13-year-old girl)*"

Most of the participants did not have positive feelings about their bodily changes. Increasing breast size, acne, appearance of pubic hair, enlargement of the nose and buttocks, vaginal secretions, back pain and dysmenorrhea as well as irregular menstrual cycles at menarche were all concerns for adolescents, the majority of whom had not previously received any education on these issues.

*“I slouch because I’m ashamed of my breasts. When guests come, I go to my room and don’t come out until they leave. I feel bad when the grownups talk about my bodily changes and tease me. I’d rather they ignore my puberty. Freckles make me ugly; I have to cover them with makeup.” (17-year-old girl)*"

*“Back pain and headaches during menstruation worry me. I usually talk about them with my friends, but they are unable to offer any help. They’re just like me.” (13-year-old girl)*"

Adolescents who took part in this study were interested in acquiring more accurate information on these issues. A minority of participants regarded these changes as positive and expressed happiness about them.

The events that participants of this study were most concerned about included vaginal discharge, pain during the menstrual period and irregular menses. In addition, a number of adolescents considered menstruation to be a sickness:

“I can’t work out when I get sick” (or would avoid taking a shower). “Mom says don’t take a shower when you’re menstruating. If that area gets soaked it will get infected, and your pain will get even worse.” *(15-year-old girl)*

#### Theme 3: Psychological changes

The majority of participants experienced psychological changes associated with puberty that included numerous codes such as sorrow, impatience, shyness, daydreaming and hunger for affection.

“I became depressed during puberty, which had a bad effect on my performance in secondary school. I‘m impatient during my menstrual period. I’ve become more embarrassed when attending social events such as parties.” (13-year-old girl)

#### Theme 4: Discord with parents

Communicating with parents was reported as problematic by the majority of participants. Extracted codes in this regard included parents failing to recognize adolescents’ concerns as well as adolescents not complying with parental advice, beginning to want independence, lacking trust in the family, disagreeing with parents’ opinions, feeling confused about their role and preferring to be with peers.

“My parents behave as if they were never teenagers. They keep picking on me for dressing like this or behaving like that. My parents keep on giving me advice, which makes me sick. I just look at them and don’t listen. Although my parents know my friends, they won’t allow me to go to their homes or to the cinema or park with them. They argue that it’s still too soon for me to go out without my family.” (18-year-old girl)

Most of the participants complained about parents exercising strict control on their personal activities such as choosing clothes, friends, and subjects to study.

“When I go shopping with my mom we like different styles. On these occasions, I either don’t choose anything or assert myself.” (13-year-old girl)

However, a minority of participants did not have a problem in this regard.

”I have no problem with my mom’s style in shopping. She is educated and a social person and knows well what’s suitable for girls my age. Her choice is also mine.” (13-year-old girl)

Confusion with one’s role as an adult or a child was also a cause of concern for some adolescents.

“When I want to go out with my friends, my mom says I’m still a child! But when it comes to running household errands, I’m a grown up! I’m confused!” (13-year-old girl)

Most of the participants showed a preference for associating with peers and enjoyed socializing with their friends.

*“Sometimes I wish I could live with my friends. They never advise me and understand my situation.” (18-year-old girl)*"

#### Theme 5: Sexual orientation and the need for education on this issue

This concept included several codes such as sexual curiosity, interest in a relationship with the opposite sex and need for correct information on sexual health.

“I’d like to know a lot about boys. I’d like to have an intimate relationship with a boy, but my mom says I shouldn’t believe what they say about love! It’s just a whim. They will eventually dump you, and you will get depressed.” (20-year-old girl)

*“I’d like to get more information on reproduction, STDs and risky sexual practices,* etc.*, but our teachers are not explicit, and we’re not comfortable asking about these things at home. So I usually get my information from my friends, the Internet, magazines or books.” (13-year-old girl)*

The adolescents who participated in this study also suggested that a school subject on sexual issues and puberty would be conducive to broadening their insights. They numbered their pubertal concerns in order of priority as relationships with the opposite sex, sexual practices, marriage, discord with parents and problems at school.

The most popular sources of information on sex for adolescents in this study were friends, Internet, magazines and books. They did not feel comfortable enough to raise these issues at home or thought their parents did not know much more than they did.

#### Theme 6: Scholastic dysfunction

Scholastic dysfunction was reported by the majority of adolescents who took part in this study, as indicated by this 13-year-old girl:

“I’ve lost my respect for education since I entered secondary school. My grades are dropping, with everything interesting to me except my studies. I’d rather spend time with my friends and go out with them, read romances and play some music. My family presses me too much about my studies. They keep on insisting that I should study this at school or that at the university. They compare me with another girl with outstanding scholastic achievements who is the pride of her family! What has she got that I don’t have?”

A 17-year-old participant stated this:

“In high school I feel better than I did in guidance school. I’m now used to puberty, and I’m more competitive.”

Being compared with peers was one of the unpleasant experiences touched on in most units of analysis. One adolescent 15 years old expressed the following opinion:

*“Parents should compare us to our own past rather than to others. They can, for example, question us about why our school performance has deteriorated.”*"

#### Theme 7: Religious considerations

Most participants expressed their need for having faith while undergoing pubescent experiences. Some girls stated that they were unaware of religious rules at the time of menstruation because they did not pay enough attention when those were taught by their teachers. However, now they feel they needed to know more about them. A participant 12 years old admitted this:

“I don’t know how or when I wash my body after bleeding or when I am allowed to enter the mosque and perform daily prayers.”

### Discussion

The point of view held by adolescent girls is of great importance in understanding and assessing their needs in puberty. Most participants in this study did not have a pleasant puberty experience. They regarded it as a troublesome time, consistent with results of other studies conducted both inside and outside Iran [5,10].

Anxiety and shame about physical appearance and psychological changes during puberty was a cause of concern for this study’s participants. Many adolescent girls are preoccupied with their appearance and may spend most of their time concerned with makeup and beauty. Embarrassment about sexual bodily changes exists in most cultures [9]. A study in Oman showed that only half of 1,675 guidance school girls were aware of bodily and sexual changes they undergo at puberty [15]. These changes may be a potential source of confusion and pressure for adolescents [1].

Similar to previous studies, this study showed that adolescent girls often do not receive accurate information about menstrual health because of culturally specific practices that lead to incorrect and unhealthy behaviors. However, educational interventions, such as the health promotion project in this study, can be quite effective in promoting menstrual health. [16,17].

One of the most annoying experiences reported by participants in this study was discord with parents. We assume this could be the result of a generation gap [18]. As extracted from the participants’ statements, lack of compliance with parental advice is a cause of discord. Findings in this study showed that adolescents tend to resist their parents’ commands. They prefer to consult their parents rather than taking advice or orders from them. Adolescents will talk about themselves with their parents provided they perceive some parental enthusiasm for their ideas [18].

Menarche in the majority of girls was unexpected and worrisome, which depicts girls’ paucity of knowledge in this area. A previous study in Iran reported that only 17.4% of the students were knowledgeable enough to embrace puberty [2]. That study also reported that 69% of the girls divulged their first menarche experience to their mother.

A related qualitative study in the US used in-depth interviews with 22 girls of different ethnic backgrounds who were between 14–18 years of age. Findings showed that those who had previously been educated to be prepared for pubertal changes coped better with them [19].

Although similar results were found in the present study, many participants were too embarrassed to get more information on these issues from their mothers. Instead, they kept some distance from their mothers to avoid puberty-related discussions. We argue that girls are often not intimate enough with their mothers to comfortably discuss their problems with them. During one study, researchers asked 157 girls in 9th grade to give their opinions on how best to prepare girls for menarche. Of them, 35% stated that adolescents need emotional support and reassurance that menstruation is a natural phenomenon and not a calamity that causes fear, anxiety and shame. Mothers can reassure their daughters that their experiences are quite normal and give them more accurate information about menstruation. The best time for such counseling sessions between parents and girls is before the onset of menarche [20].

It is possible, to some extent, to correct existing misinformation about menstruation. For example, we could include anatomy lessons in the school curriculum to eliminate adolescents’ common misunderstandings [21]. A study in Iran stipulated that the most efficient strategy to convey the necessary information to girls is by involvement of families, and mothers in particular [22].

A tendency towards independence in adolescence is another cause for discord between parents and children. It is part of adolescents’ nature to oppose any dominant source of power within their social circle, including parents. Parents and adolescents may have contradictory ideas about various issues that at times may result in discord. Parents tend to regard handing responsibilities over to their teenaged children as a risk, and thus they try not to lose control over their teenagers’ decision making or problem solving process. Adolescents may ask “What kind of independence am I getting when everybody is telling me what or what not to do?” Parents may find striking a balance between exercising their authority and respecting their children’s freedom quite a challenge [23].

As mentioned, adolescents prefer to socialize with their peers rather than their families, which may be the reason they are less likely to be critical or assertive around friends. Previous studies have shown that protective parents who fail to establish an intimate relationship with their teenaged children tend to harbor a negative opinion about their children’s best friends, which may be a source of mistrust and distance between parents and children. Teenagers who have good interaction with each other enjoy a higher level of integrity and do better at school. Parents should encourage their adolescent children to have social gatherings at their own homes so they can meet their friends [24].

It is quite common for adolescents to experience a dip in their school performance, attributable to new activities in the teenage years [24]. Most of the adolescents who participated in this study experienced a loss of motivation for studying. Parents who make comparisons between their adolescent and other adolescents contribute to possible emotional problems in their child that may lead to educational frustration. Rather than comparing their adolescent with peers, parents should encourage their child to make friends with other adolescents, or according to one participant, they should compare each teenager to his or her own past rather than other adolescents [24].

Sexual drive and adolescents’ lack of proper information in this regard was one of the highest ranking concerns reported by most participants. The majority wanted to know more about topics such as STDs, naive and high risk sexual practices and reproduction. A previous study showed that Tehrani adolescent girls’ priorities in sexual education included pubertal health and healthy psychological maturity [25].

Despite adolescent girls’ need and desire to learn more about sexual issues, their mothers may expect them to remain naïve and unaware of these “shameful” topics [5]. It should be reiterated that addressing the needs of girls is an investment in the future of a country. According to previously conducted studies in Iran, the number of adolescents who date the opposite sex is 3 times more than in the past. Their relationships have progressed beyond friendship to having sex, and the number of adolescents with multiple sexual partners is increasing. This trend, rooted in the adolescent sexual and psychological revolution, is introducing a host of pathologies in adolescence. Islam has by no means made sex taboo or prohibited education regarding sexual issues. Islamic literature contains modules on sexual education. In fact, it is a must for Muslims to learn appropriate sexual practices [26], with failure to address such educational needs invariably associated with problems such as unwanted pregnancies and STDs [9].

A qualitative study in Iran concluded that in view of the Iranian cultural and religious background, families and religious beliefs play a pivotal role in reducing high risk sexual behaviors in Iranian adolescents [27]. Nonetheless, the importance of the teachers’ role in maintaining ethical values can never be overestimated [26].

Most adolescents in this study reported that they knew very little about the implications of Islamic and Sharia rules on ritual washing, praying and fasting after puberty and during menstruation. However, after their first experience of menstruation they felt they needed to know more about them. A previous study in Mazandaran, Iran showed that although almost 76% of students knew the correct answers to questions about menstruation and Sharia practices, only 56% knew the correct method of ablution during the menstrual period [5]. A study in Isfahan, Iran reported that implications of Sharia rules with regard to pubertal changes comprise an important entity [28].

Finally, the importance of collaboration between adolescents, their families and society to broaden insights on puberty and its essential needs should be reiterated.

#### Limitations

This study has some limitations that must be acknowledged. Teenagers' sense of shame could have impeded a deepening of the interviews. In addition, adolescents in this study were from one city in Iran. Only a limited number of interviews were conducted and limited information collected, and thus the small sample is not necessarily representative of all female Iranian teenagers. Some teens who live in the capital of Tehran may have a different level of awareness about menstruation than teens living in different parts of Iran, based on various levels of social, personal, and environmental factors. Furthermore, fear and shame associated with sharing and the nature of data collection at the time of the interviews, with respect to the age of the participants, may have limited open sharing about menstruation among the young participants. Further research needs to be conducted in different parts of Iran to extend the insights and findings of this study.

### Conclusion

Findings from the current study show that for the majority of the participants, puberty was an unpleasant experience. Most of them were in need of education regarding how to address issues surrounding puberty. The society, families and of course the adolescents themselves are responsible for working together to create an atmosphere in which correct information on puberty and the associated issues is readily accessible.

### Competing interests

The author(s) declare that they have no competing interests'.

### Author contributions

ZH, NAHG, and MF designed the study, NAHG, ZH, and LH collected and analyzed data. NAHG and ZH wrote the first draft of the manuscript, which has been commented on by the other authors. Both authors contributed to and approved the final version of the manuscript.

## Pre-publication history

The pre-publication history for this paper can be accessed here:

http://www.biomedcentral.com/1471-2458/12/698/prepub
